# Structural Characteristics of Mitochondrial Genomes of Two Species of Mackerel and Phylogenetic Analysis of Scombridae Family

**DOI:** 10.3390/biom15040555

**Published:** 2025-04-09

**Authors:** Jianqi Yang, Ang Li, Shufang Liu

**Affiliations:** 1 State Key Laboratory of Mariculture Biobreeding and Sustainable Goods, Yellow Sea Fisheries Research Institute, Chinese Academy of Fishery Sciences, Qingdao 266071, China; 2 College of Fisheries and Life Sciences, Dalian Ocean University, Dalian 116023, China; 3 Laboratory for Marine Fisheries Science and Food Production Processes, Qingdao Marine Science and Technology Center, Qingdao 266237, China

**Keywords:** *Scomberomorus guttatus*, *Scomberomorus commerson*, mitochondrial genome, sequence analysis, systematic evolution

## Abstract

*Scomberomorus guttatus* and *Scomberomorus commerson* are both important marine economic fish species worldwide, with high scientific and ecological value. In this study, the complete mitochondrial genome sequences of these two species of mackerel were obtained by using next-generation sequencing technology, with total lengths of 16,562 bp and 16,594 bp, respectively. Like most teleosts, both species possess 13 protein-coding genes, 22 *tRNA* genes, 2 *rRNA* genes, and 1 non-coding region *D-loop*. The base composition showed significant AT bias (55.1%, 53.4%) and anti-G bias (16.0%, 16.2%). In their control area, the terminal-associated sequence (TAS) was identified, and a total of three core sequences with repeated “---TACAT---ATGTA---” were found. There are typical CSB-E structures and CSB-D-like structures in the central conserved domain (CD), but no CSB-F structures have been found. Meanwhile, the CSB-2 and CSB-3 structures were identified in the conserved sequence block (CSB), but the CSB-1 structure was missing. To further investigate the phylogenetic relationships within the Scombridae family, this study conducted a comparative analysis of mitochondrial genomes from 30 Scombridae species. Phylogenetic trees encompassing 60% of the documented Scombridae species were constructed using the Neighbor-Joining (NJ) and Maximum Likelihood (ML) methods. The results revealed a close evolutionary relationship between the genus *Scomber* and *Rastrelliger*, while the genus *Scomberomorus* exhibited closer affinities to *Thunnus*, *Euthynnus*, and *Katsuwonus*. At the species level, *Scomberomorus guttatus* diverged earlier from *Scomberomorus commerson*. These findings refine and update the phylogenetic relationships among Scombridae species, providing critical molecular evidence and insights for deeper exploration of their evolutionary history and genetic affinities.

## 1. Introduction

*Scomberomorus guttatus* and *Scomberomorus commerson*, both belonging to the family Scombridae of the order Scombriformes, are important marine economic fish species worldwide. They are widely distributed in the seas around the Korean Peninsula, Japan, and the Indo-West Pacific continental shelf. Research by domestic and foreign scholars on *S. guttatus* and *S. commerson* has mainly focused on morphological, growth, reproductive, and dietary characteristics, as well as ecological features such as migration and distribution, with relatively little attention given to their genetics [1]. Molecular genetic data provide crucial evidence for elucidating the population’s genetic structure and evolutionary relationships [2]. However, there has been no study on the complete mitochondrial genome sequences of *S. guttatus* and *S. commerson*.

Currently, the family Scombridae comprises 15 genera and 51 species [3]. Research on species within this family has primarily focused on population structure, stock identification, and phylogeography [4,5,6,7], with relatively less attention paid to systematic classification and evolution. Only a few scholars have previously analyzed their phylogenetic relationships based on single gene fragments [8,9,10]. However, single-gene fragments can only provide limited genetic information and fail to fully reflect the genetic diversity and evolutionary history. The resulting phylogenetic trees typically exhibit low resolution, making it difficult to accurately distinguish closely related species or reveal subtle differentiation within species. Additionally, taxonomic controversies persist regarding the genus *Thunnus* [11,12], and certain species identification issues remain within Scombridae [13]. Therefore, analyzing the phylogenetic relationships of Scombridae using mitochondrial genome protein-coding sequences holds significant scientific importance.

Mitochondrial DNA (mtDNA) is a circular, double-stranded, independent genome of approximately 15–20 kb in length, distinct from the nuclear genome. Due to its maternal inheritance, simple structure, ability for independent replication, high mutation rate, and relatively stable probability of occurrence of variations, mtDNA has been widely applied in studies of species evolution, phylogenetic relationships, and population genetics. It has become a powerful tool for resolving genetic differentiation among closely related species and populations [14,15,16]. In recent years, advancements in DNA sequencing technologies have greatly facilitated the rapid and accurate acquisition of mitochondrial genome data for fish species. Consequently, an increasing number of complete mitochondrial DNA sequences of fish have been reported [17,18,19,20]. This study determined and analyzed the complete mitochondrial genome sequences of *S. guttatus* and *S. commerson*. By comparing these sequences with those of 30 other species within the Scombridae family, we further explored the molecular phylogenetic relationships of Scombridae fish. The results of this research aim to provide basic information for the genetic diversity of *Scomberomorus* genus and the phylogenetic relationships of Scombridae family.

## 2. Materials and Methods

### 2.1. Experimental Materials and DNA Extraction

The samples of *S. guttatus* and *S. commerson* used in this study were obtained from the National Marine Fisheries Bioresource Repository’s fish DNA barcode voucher specimen library. Partial dorsal muscle tissue was excised and placed in 1.5 mL EP centrifuge tubes, fixed in 95% ethanol for 48 h, and then stored at −20 °C in a freezer for subsequent use. Genomic DNA was extracted from the tissue samples using the marine organism genomic DNA extraction kit from Beijing Tiangen Biotechnology Co., Ltd., China. DNA integrity was assessed by 1% agarose gel electrophoresis.

### 2.2. Sequencing, Gene Annotation, and Analysis

We commissioned Wuhan BGI to perform mitochondrial whole genome sequencing. The mitochondrial genome was extracted and assembled using NOVOPlasty 4.3 software [21]. Between October and November 2023, the complete mitochondrial genome sequence obtained from the assembly was uploaded to the MITOS web server (http://mitofish.aori.u-tokyo.ac.jp/, accessed on 15 October 2023) for annotation, with the reference sequences of closely related species, Australian Spanish mackerel (*Scomberomorus munroi*: JX559743.1) and Barred Spanish mackerel (*Scomberomorus semifasciatus*: NC_021391.1). The final positions and lengths of each gene were determined and verified through manual alignment.

We used the online tRNAscan-SE 2.0 software (http://trna.ucsc.edu/tRNAscan-SE/, accessed on 15 October 2023) [22] to predict the secondary structure of tRNA genes. MEGA 7 software [23] was employed to analyze base composition and codon usage, calculate AT-skew and GC-skew values, and graphical analysis was conducted using Microsoft Excel software (https://www.microsoft.com/en-us/microsoft-365/excel, accessed on 15 October 2023).

### 2.3. Phylogenetic Analysis

In addition to the two new mitochondrial genomes obtained in this study, the GenBank database also records mitochondrial genome sequences of 30 species from 11 genera in the family Scombridae (Table 1). We extracted the mitochondrial genome protein-coding sequences of all mackerel fish, with *Siniperca chuatsi* and *Girella punctate* as outgroups. We used the Kimura-2-parameter model of Mega7.0 software to construct Neighbor-Joining (NJ) and Maximum Likelihood (ML) phylogenetic trees, respectively.

## 3. Results and Analysis

### 3.1. Composition and Localization of Mitochondrial Genome

**Figure 1:**
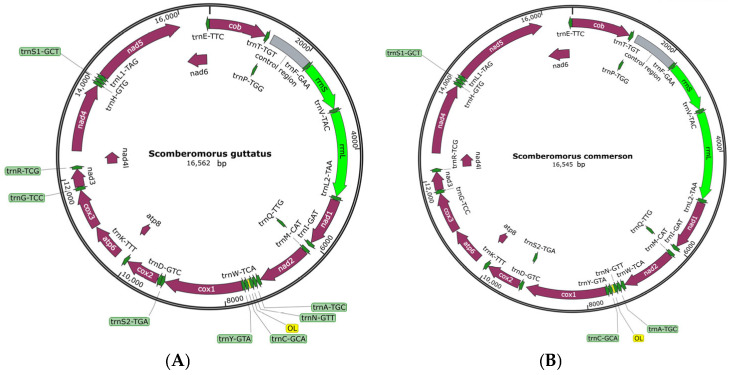
Mitochondrial Genome Structure of *S. guttatus* (**A**) and *S. commerson* (**B**).

Analysis of the mitochondrial genomes of *S. guttatus* and *S. commerson* showed that their total lengths are 16,562 bp and 16,545 bp (Figure 1), respectively, comprising 37 genes and non-coding regions. The structure is highly conserved, with the types and arrangement of each gene conforming to the typical structure of vertebrate mitochondrial genomes (Table 2). Specifically, they include 13 protein-coding genes (*PCGs*); 7 NADH dehydrogenase subunits (*ND1*, *ND2*, *ND3*, *ND4*, *ND4L*, *ND5*, *ND6*); 3 cytochrome c oxidase subunits (*COI*, *COII*, *COIII*); 2 ATP synthase subunits (*ATP6*, *ATP8*); 1 cytochrome b gene (*Cytb*); 2 rRNA genes (*12S rRNA*, *16S rRNA*); and 1 control region (*D-loop*). The non-coding region mainly consists of two parts: the control region and the light strand replication origin.

### 3.2. Analysis of Mitochondrial Genome Base Composition

Based on the analysis of nucleotide composition of the mitochondrial genome sequences of *S. guttatus* and *S. commerson*, it was observed that the A + T base content was significantly higher than G + C, indicating a pronounced AT bias (55.1% and 53.5%, respectively). Additionally, both species showed low guanine (G) content (16.0% and 16.2%, respectively), indicating a significant anti-G bias in the base composition of their mitochondrial genomes. This anti-G bias was particularly prominent in protein-coding genes, with G content only 15.3% and 15.6%, respectively (Table 3).

### 3.3. Analysis of Gene Segments in Mitochondrial Genome

#### 3.3.1. Base Composition and Content of Protein-Coding Genes

**Figure 2:**
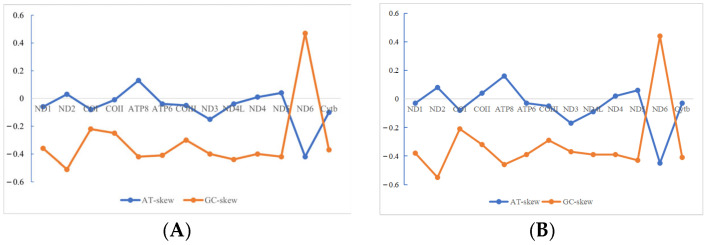
The distribution of biases in protein-coding genes of the mitochondrial genomes of *S. guttatus* (**A**) and *S. commerson* (**B**).

The distribution of bases in protein-coding genes of mitochondrial genomes of *S. guttatus* and *S. commerson* is uneven. Specifically, the highest T base content is observed in *ND6* located on the L chain (39.5% and 39.1%), while the lowest is in *ATP8* (23.8% and 22.6%); the highest C base content is in *ND4L* (35.7%), while the lowest is in *COII* (26.6%); the highest A base content is in *ATP8* (31.0%), while the lowest is in *ND6* (16.1% and 14.8%); the highest G base content is in *ND6* (32.6% and 33.3%), while the lowest is in *ND2* (11.3% and 10.6%). Regarding the base composition of codons, the four bases at the first position of codons are relatively similar in content, indicating no clear preference for base usage. At the second position of codons, the T base content is highest (35%), while the G base content is lowest (14.9%), indicating a significant T bias and anti-G bias. At the third position of codons, the C base content is highest (32.4%), while the G base content is lowest (11.2%). In summary, the mitochondrial protein-encoding gene *ND6* of both *S. guttatus* and *S. commerson* exhibited a strong GC bias in the mitochondrial protein-coding gene *ND6*, while the rest of the genes showed an AT bias (Figure 2).

**Figure 3:**
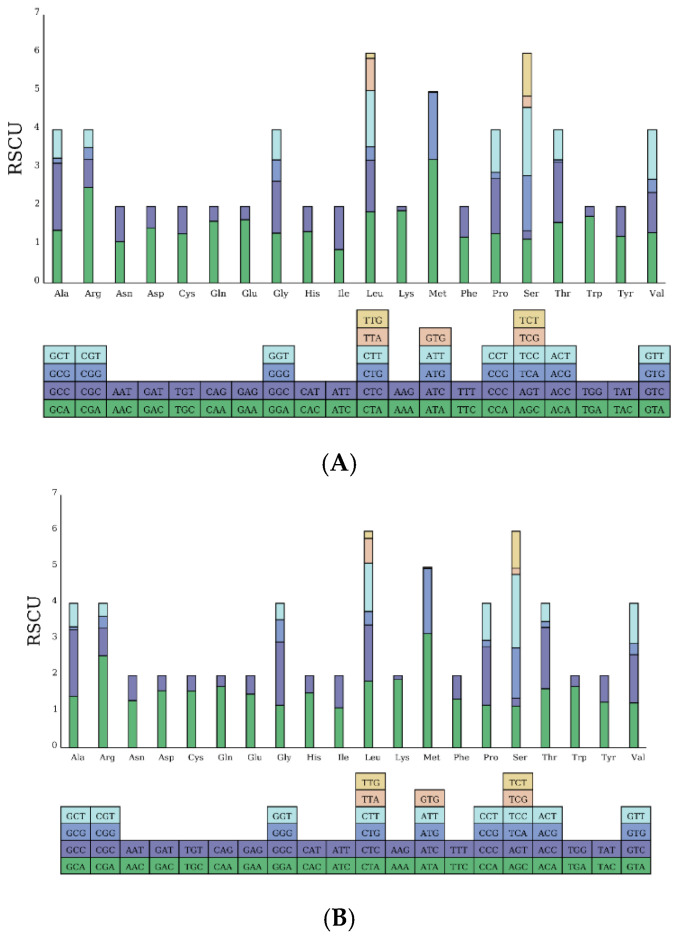
Amino acid types and quantities in protein-coding genes of *S. guttatus* (**A**) and *S. commerson* (**B**).

A preliminary analysis of the relative synonymous codon usage (RSCU) and the types and quantities of amino acids of the 13 protein-coding genes in the mitochondrial genome was conducted. The results show that both species encode a total of 3812 and 3816 amino acids, with Leucine being the most frequently used amino acid (17.23%, 17.39%), and termination codons being the least frequent, accounting for 0.34% each (Figure 3). There are 34 preferred codons (RSCU ≥ 1) among the 13 protein-coding genes. For *S. guttatus*, the codon UAA, encoding Leucine, is the most common (RSCU = 3.6924), followed by AUA, encoding Methionine (RSCU = 3.225). While for *S. commerson*, the codon AUA, encoding Methionine, is the most common (RSCU = 3.1595), followed by CGA, encoding Arginine (RSCU = 2.5456).

#### 3.3.2. *rRNA* and *tRNA* Structural Features

The 12s rRNA gene in *S. guttatus* and *S. commerson* is between *tRNA-Phe* and *tRNA-Val*, while the *16S rRNA* gene is between *tRNA-Val* and *tRNA-Leu*. The H chain encodes both and has no intergenic or overlapping regions. The AT content of rRNAs is 53.8% and 53.7%, respectively, while the GC content is 46.2% and 46.3%, with A bases being the most prevalent at 31.3% and 32.2%, respectively (Table 3). The secondary structure of *rRNA* genes forms several stem-loop structures, with a predicted free energy of −259.70 kcal/mol for *12S rRNA* and −433.20 kcal/mol for *16S rRNA* in S. guttatus. In S. commerson, the predicted free energy is −255.30 kcal/mol for *12S rRNA* and −269.52 kcal/mol for *16S rRNA*.

**Figure 4:**
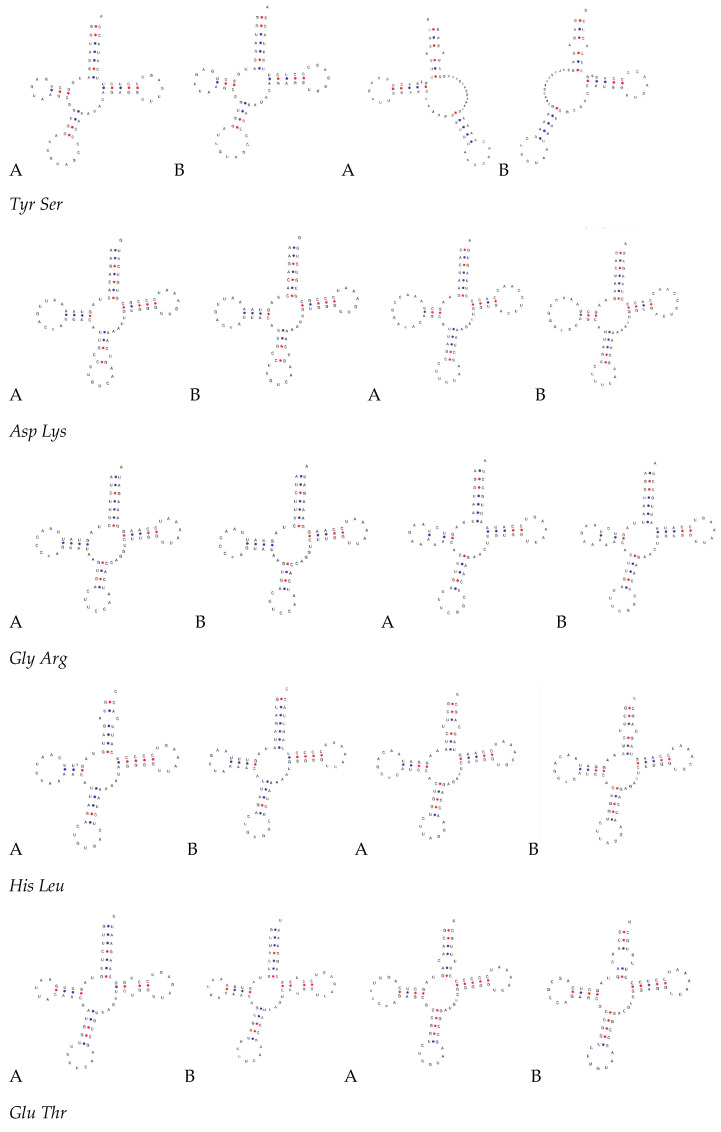

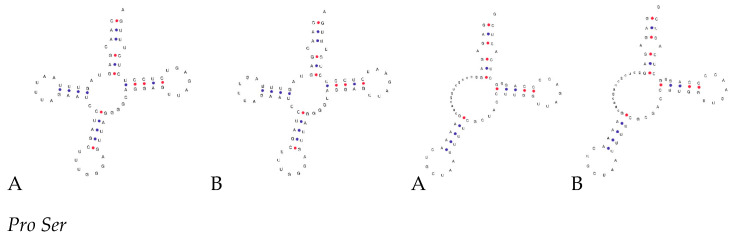
Predicted secondary structures of the 22 tRNAs in *S. guttatus* (**A**) and *S. commerson* (**B**) mitochondrial genomes.

Both *S. guttatus* and *S. commerson* mtDNA contains 22 *tRNA* genes ranging in length from 65 to 76 bp. These tRNAs are encoded on both the H chain and the L chain, with *tRNA-Pro*, *tRNA-Gln*, *tRNA-Ala*, *tRNA-Asn*, *tRNA-Cys*, *tRNA-Tyr*, *tRNA-Ser* (TGA), and *tRNA-Glu* located on the L chain, while the remaining 14 tRNAs are encoded on the H chain (Table 2). The AT content of *tRNA* genes in *S. guttatus* and *S. commerson* is 54.7% and 53.9%, respectively, while the GC content is 45.3% and 46.0%, respectively (Table 3). The *tRNA-Ser* lacks the dihydrouridine (DHU) arm, forming only a 12 bp loop, a structure commonly observed in vertebrates. The remaining 21 tRNAs can fold into typical cloverleaf secondary structures (Figure 4), each comprising multiple stem-loop structures, including the aminoacyl acceptor arm, aminoacyl acceptor loop, anticodon arm, anticodon loop, TYC arm, TV loop, DHU arm, DHU loop, and aminoacyl acceptor arm.

Comparative analysis of the sequences and structures of various tRNA genes reveals that only six *tRNA* genes—*tRNA-Met*, *tRNA-Ala*, *tRNA-Cys*, *tRNA-Asp*, *tRNA-Gly*, and *tRNA-Glu*—underwent mutations between *S. guttatus* and *S. commerson*. Each gene had only one mutation site, which did not affect the formation of their secondary structures and did not occur in the anticodon’s three bases. In the *tRNA* gene structure of *S. guttatus*, a total of 13 pairs of non-paired bases were detected, involving six types of base mismatches: A-C, A-A, A-G, C-C, U-C, and U-U mismatches. Among these 13 pairs of mismatched bases, 10 pairs occurred in the aminoacyl acceptor arm, involving *tRNA-Phe*, *tRNA-Leu*, *tRNA-Ile*, *tRNA-Trp*, *tRNA-Ser*, *tRNA-His*, *tRNA-Leu*, *tRNA-Thr*, *tRNA-Pro*, and *tRNA-Ser*. One pair occurred in the anticodon arm of *tRNA-Met*, one pair in the DHU arm of *tRNA-Arg*, and one pair in the TψC arm of *tRNA-Glu*. Such mismatched bases mainly occur in the aminoacyl acceptor arm, a phenomenon observed in other fish species as well. Some mismatches can be corrected through later *RNA* editing, without causing amino acid transport disorders [24].

#### 3.3.3. Structural Features of the Mitochondrial Genome Control Region

**Figure 5:**
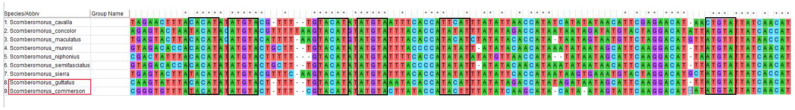
The analysis of the mitochondrial *D-loop* structure components in *S. guttatus* and *S. commerson*.

The mitochondrial control region (*D-loop*) is a non-coding region of mitochondrial DNA with the fastest evolutionary rate, and is therefore widely used in population genetics and molecular systematics studies of fish. In the mitochondrial genomes of *S. guttatu* and *S. commerson*, the control region is located between *tRNA-Pro* and *tRNA-Phe*, with a length of 857bp. The A (32.2%, 31.1%) and T (30.9%, 32.1%) base contents in the *D-loop* region are relatively high, while the C (20.9%, 22.0%) and G (16%, 14.9%) base contents are relatively low. From the 5′ end adjacent to *tRNA-Pro*, it can be divided into the terminal-associated sequence (TAS), the central conserved domain (CD), and the conserved sequence block (CSB). The research revealed certain differences in the position, quantity, and base composition of these regions between *S. guttatu* and *S. commerson* (Figure 5).

##### Termination-Associated Sequence Region

Liu Huanzhang [25] pointed out that the main structure of fish extended termination-related sequences is formed by “---TACAT---ATGTA---”. In this research, via sequence alignment of closely related fish species within the genus *Scomberomorus*, we have identified three recurring core sequences of “---TACAT---ATGTA---” in the control region sequences of both *S. guttatu* and *S. commerson*. Additionally, the control region sequences of other closely related species within the *Scomberomorus* genus also encompass these three recurring core sequences of “---TACAT---ATGTA---”.

##### Central Conserved Domain Translation

This study revealed that *S. guttatu* and *S. commerson* exhibit typical CSB-E and CSB-D structures. Notably, the CSB-E critical sequence contains a GTGGG-box, while no CSB-F structure was identified. Due to variations in the central conserved domain among fish species, some species rarely have all three structures of CSB-D, CSB-E, and CSB-F simultaneously. Lee et al. [26] analyzed the mitochondrial control regions of 23 species from 6 genera of fish and only identified the presence of CSB-D. Similarly, Zeng et al. [27] only identified CSB-D in the mitochondrial control region of the large yellow croaker.

##### The Conservative Sequence Region

In this study, CSB-2 and CSB-3 structures were identified in the conservative sequence region, while the CSB-1 structure was not recognized. CSB-2 and CSB-3 were identified as 5ʹ-TAAACCCCCCTACCCCCC-3ʹ and 5ʹ-TGTAAACCCCCCGTAAACA-3ʹ, respectively. By comparing the fish control region sequences with those of *S. guttatus* and *S. commerson*, it was found that CSB-2 is the most conservative among fish species, with nearly identical sequences containing two segments of C repeats interspaced by two–three pyrimidine nucleotides. CSB-3 exhibits greater variation among fish species and is difficult to identify. Studies have indicated its association with the replication origin of mitochondria, particularly in mammals, where CSB-1 is highly conserved. However, in fish, CSB-1 shows the greatest variability within the conservative sequence region. The phenomenon is also observed in other fish species [28].

### 3.4. Results of Phylogenetic Analysis

**Figure 6:**
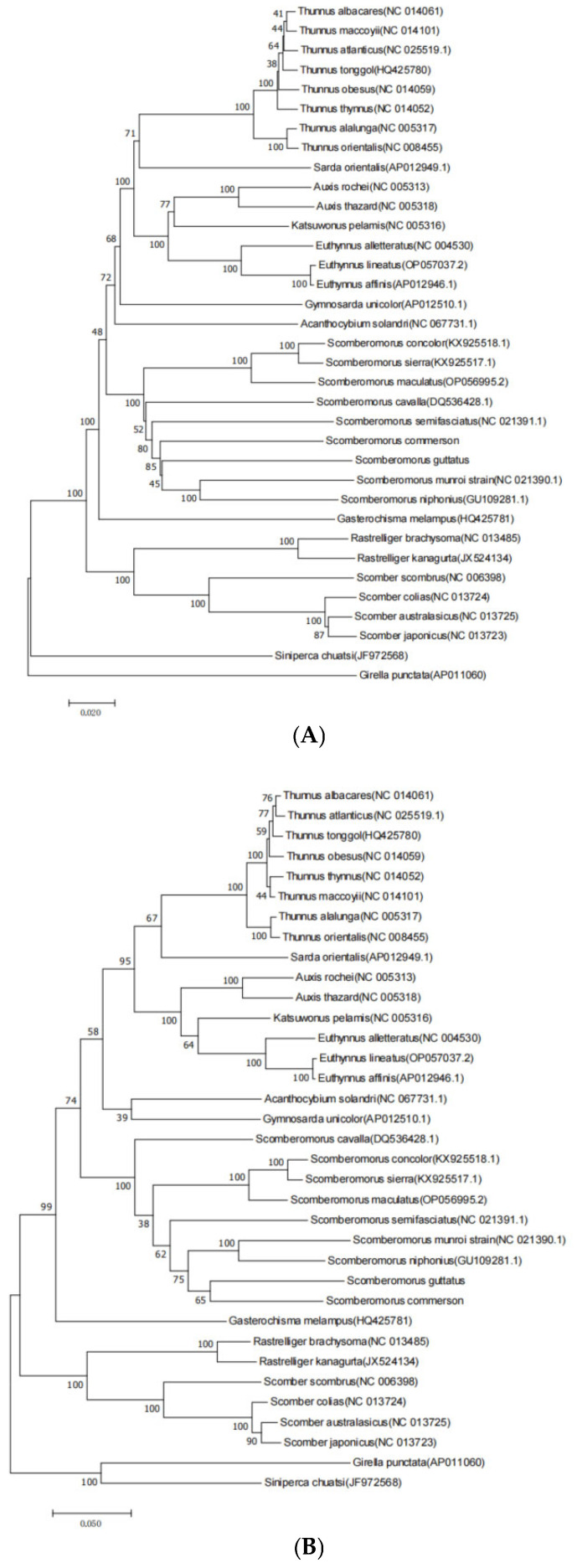
Molecular phylogenetic trees based on concatenated sequences of 12 PCGs constructed using NJ (**A**) and ML (**B**) methods.

The constructed phylogenetic tree is shown in Figure 6. Both phylogenetic trees clearly divide the 32 species of Scombridae into two clades: one cluster includes the genus *Scomber* and the genus *Rastrelliger*, while the other cluster comprises the remaining species including the genera *Thunnus*, *Katsuwonus*, *Auxis*, *Euthynnus*, *Gymnosarda*, *Acanthocybium*, *Sarda*, and *Scomberomorus*. Clustering of species in the same genus is more effective. The NJ phylogenetic tree constructed using protein-coding sequences shows slightly lower confidence values in some evolutionary branches compared to the ML phylogenetic tree. The genus *Scomber* forms a monophyletic group, with relatively close phylogenetic relationships to the tribe *Thunnini* compared to the genus Scomberomorus. Both NJ and ML phylogenetic trees indicate that within the genus *Scomberomorus*, *S. niphonius* and the *S. munroi* form one cluster, while the *S. concolor*, *S. sierra*, and *S. maculatus* form another cluster, with the divergence time between the *S. guttatus* and *S. commerson* being relatively early within the genus *Scomberomorus*. Within the tribe *Thunnini*, the genera *Thunnus*, *Auxis*, and *Euthynnus* are sister groups. The genus *Thunnus* is divided into two evolutionary branches: one branch includes *T.alalunga* and *T.orientalis*, while the other branch comprises *T. albacares*, *T. atlanticus*, *T. maccoyii*, *T. obesus*, *T. thynnus*, and *T. tonggol*. Unlike the NJ phylogenetic tree, the ML phylogenetic tree shows that *T. thynnus* and *T. maccoyii* form a separate cluster; in the NJ phylogenetic tree, *T. albacares* clusters with *T. maccoyii*, while in the ML phylogenetic tree, *T. albacares* clusters with *T. atlanticus*.

## 4. Discussion

This study employed next-generation sequencing technology to obtain the complete mitochondrial genome sequences of *S. guttatus* and *S. commerson*. An analysis of the mitochondrial DNA gene structures of these two species was conducted. Additionally, the molecular phylogenetic relationships within the Scombridae family were investigated using concatenated sequences of 12 protein-coding genes (PCGs) from 32 species of mackerel-like fishes, employing Neighbor-Joining (NJ) and Maximum Likelihood (ML) methods.

### 4.1. Mitochondrial DNA Sequence Structural Features of S. guttatus and S. commerson

The mitochondrial DNA sequences of both *S. guttatus* and *S. commerson* exhibit relatively high A + T content and low G + C content, demonstrating a pronounced AT bias. This phenomenon is also observed in other fish species, albeit with slight variations in content due to species differences. The underlying reasons for this base preference are likely associated with natural mutations and selection pressures during replication and transcription processes [29].

The protein-coding genes of both species, except for the first codon position, exhibit a relatively uniform distribution of each base. However, the second and third codon positions show a distinct bias against G, indicating a preference for other nucleotides. The restrictive influence of amino acids on base distribution and the corresponding differences in codon usage frequency are important factors contributing to the uneven distribution of bases in protein-coding genes [30]. Among the 20 amino acids encoded, Leucine (Leu) is the most frequently utilized, a phenomenon commonly observed in other fish species as well [31,32]. This study found that the absolute value of the free energy of the *12S rRNA* gene is lower than that of the *16S rRNA* gene. Generally, lower free energy values indicate greater molecular stability, suggesting that the former is more conservative than the latter. Additionally, the secondary structure prediction of the *16S rRNA* gene also reveals more frequent nucleotide substitutions, insertions, and rearrangements, indicating a higher degree of variation compared to the *12S rRNA* gene. Therefore, the *12S rRNA* gene is often used as a DNA barcode for fish identification and phylogenetic studies [33].

Among the 22 tRNAs, both species exhibit a *tRNA-Ser* that cannot form a cloverleaf structure, a common feature in the mitochondrial gene genomes of most vertebrates, including fish. Studies have shown that such tRNAs lacking the DHU arm can adjust their structure and function later to integrate into the ribosome, facilitating their role in carrying and transporting amino acids [34]. Additionally, the phenomenon of base mismatches is widespread in the secondary structure of tRNAs. Since the mitochondrial genome is not influenced by the recombination process, base mismatches are allowed, and these mismatches help eliminate harmful mutations [35]. The mitochondrial control region primarily comprises regulatory elements controlling mitochondrial genome replication and expression. Investigating its structural and functional aspects will contribute to understanding DNA replication, transcription mechanisms, and evolutionary patterns [36,37].

In the control area of *S. guttatus* and *S. commerson*, the terminal-associated sequence (TAS) was identified, and a total of three core sequences with repeated “---TACAT---ATGTA---” were found. Additionally, the control region sequences of other closely related species within the *Scomberomorus* genus also encompass these three recurring core sequences of “---TACAT---ATGTA---”. The central conserved domain exhibited a typical CSB-E structure and CSB-D structure, while no CSB-F structure was found. In the conserved sequence region, we identified CSB-2 and CSB-3 structures but failed to identify the CSB-1 structure.

### 4.2. Molecular Phylogenetic Analysis of Scombridae

Presently, phylogenetic studies on Scombridae fishes are mostly based on single genes such as mitochondrial *Cytb* or *ATP* [38,39]. However, single gene information has limitations and cannot fully elucidate the complex evolutionary history and relationships among species. The use of a multi-gene approach can more accurately reflect genetic differences and evolutionary relationships among species. Considering that only the *ND6* gene is located on the L strand, and the heterogeneity of its nucleotide composition can lead to poor performance in phylogenetic analysis [40]. Therefore, in this study, concatenated sequences of the 12 protein-coding genes encoded by the H strand were used to construct the phylogenetic tree of Scombridae based on the NJ and ML methods.

In this study, each node was labeled with support scores, which were calculated by combining data from multiple genes. Compared with the support rate based on single-gene data alone [38,39], this polygenic dataset significantly improves the support rate of key nodes, thereby enhancing the reliability and comprehensiveness of the phylogenetic tree

Chen Ying et al. [41] constructed phylogenetic relationships based on the protein-coding genes of 8 genera and 20 species of Scombridae, while our study further enriched and refined this phylogenetic framework based on mitochondrial genome sequences of 11 genera and 32 species of Scombridae. Our results showed that 6 species within the genus *Scomber* and *Rastrelliger* have close genetic relationships, as do 26 species across the genera *Seriola*, *Thunnus*, *Neothunnus*, *Katsuwonus*, *Auxis*, *Gymnosarda*, *Alepisaurus*, and *Xenisthmidae*. These findings not only support the conclusions of Chen Ying et al. [41]. but also provide more robust evidence for the phylogenetic relationships of Scombridae through a broader sample range and more comprehensive genomic data.

Compared to the studies by Jeena, Qiu Fan, and others [41,42], our results further refine the phylogenetic relationships within the genus Seriola. Their research was based on *COI* and *Cytb* gene fragments, as well as partial sequences of ITS1 for analyzing the molecular systematic evolution of Scombridae. In contrast, we utilized a more comprehensive mitochondrial genome dataset, providing higher resolution and a more accurate phylogenetic tree.

In addition, the mitochondrial genomes of *S. guttatus* and *S. commerson* have not been studied in detail. This study fills this gap, enriches the data resources of the mitochondrial genomes of Scombridae fishes, and further updates and refines the phylogenetic relationships among Scombridae fishes. Through this study, we not only deepen our understanding of the genetic diversity of Scombridae fishes, but also provide valuable basic information for future scientific research in related fields.

Although our study provides important phylogenetic information, the limited sample size may not fully represent the diversity of the entire Scombridae family. Future research should expand the sample range to obtain more comprehensive results. Future studies should focus on sequencing the mitochondrial genomes of more Scombridae species to further validate and refine our phylogenetic tree. Additionally, incorporating nuclear genomic data could provide a more comprehensive perspective.

## 5. Conclusions

In summary, this study conducted an analysis of the mitochondrial DNA genome structure of *S. guttatus* and *S. commerson*. Utilizing concatenated sequences of 12 protein-coding genes from 32 Scombridae species, the molecular phylogenetic relationships within the Scombridae family were analyzed using both Neighbor-Joining (NJ) and Maximum Likelihood (ML) methods. The main findings of the study are summarized as follows:

(1) The mitochondrial DNA sequences of spotted mackerel and king mackerel are 16,562 bp and 16,545 bp, respectively. Both species possess 13 protein-coding genes, 22 *tRNA* genes, 2 *rRNA* genes, and 1 non-coding region (*D-loop*). The base composition consists of A (28.5%, 28.1%), T (26.6%, 25.3%), G (16.0%, 16.2%), and C (28.9%, 30.3%), indicating a high A + T content and a low G + C content, demonstrating a significant AT bias. Protein-coding genes in both species exhibit a relatively uniform distribution of bases at the first codon position, while the second and third positions show a clear anti-G bias. Among the 20 amino acids encoded, Leucine (Leu) is the most frequently used. This study reveals that the absolute value of the free energy of the 12S *rRNA* gene is lower than that of the *16S rRNA* gene. In both species, the *tRNA-Ser* lacks the DHU arm, preventing the formation of a cloverleaf structure. In control area of *S. guttatus* and *S. commerson*, the terminal-associated sequence (TAS) was identified, and a total of three core sequences with repeated “---TACAT---ATGTA---” were found. In the control region of *S. guttatus* and *S. commerson*, termination signal regions, central conserved regions. The central conserved region exhibits a typical CSB-E structure and CSB-D structure, but no CSB-F structure was found. The conserved sequence block did not reveal a CSB-1 structure.
(2) In this study, a phylogenetic tree of 60% of the Scombridae fish species was constructed using concatenated sequences of 12 protein-coding genes encoded by the heavy (H) strand of the mitochondrial genome. The tree was built using the NJ and ML methods. The results revealed a close relationship between the Scomber genus and the Acanthocybium genus, while the genus Scomberomorus showed closer affinity with the genera *Thunnus*, *Auxis*, *Katsuwonus*, *Acanthocybium*, *Gymnosarda*, *Scomberomorus*, *Grammatorcynus*, and *Rastrelliger*. Furthermore, within the genus *Scomberomorus*, a monophyletic group was formed, with the spotted mackerel and the king mackerel diverging early. *S. niphonius* and the *S. munroi* formed a separate clade, whereas *S. concolor*, *S. sierra*, and *S. maculatus* were sister groups. This study provides a more comprehensive understanding of the phylogenetic relationships within the Scombridae family, further confirming the evolutionary relationships among its members.

This study enriches the mitochondrial genome information of the Scombridae family, leading to a deeper understanding of the evolutionary overview and phylogenetic relationships of Scombridae fish. These findings offer a novel perspective, prompting a deeper exploration of the mitochondrial DNA sequences and evolutionary mechanisms of Scombridae fish, facilitating a better understanding of their evolutionary history, population genetic structure, as well as their roles and functions in ecosystems.

## Figures and Tables

**Table 1:** The mitochondrial genome information of scombroid fishes used for constructing phylogenetic trees.

Genera	Species	GenBank ID
*Auxis*	*Auxis rochei*	NC_005313
	*Auxis thazard*	NC_005318
*Euthynnus*	*Euthynnus alletteratus*	NC_004530
	*Euthynnus affinis*	AP012946.1
*Gasterochisma*	*Gasterochisma melampus*	HQ425781
*Katsuwonus*	*Katsuwonus pelamis*	NC_005316
*Rastrelliger*	*Rastrelliger brachysoma*	NC_013485
	*Rastrelliger kanagurta*	JX524134
*Scomber*	*Scomber australasicus*	NC_013725
	*Scomber colias*	NC_013724
	*Scomber japonicus*	NC_013723
	*Scomber scombrus*	NC_006398
*Scomberomorus*	*Scomberomorus cavalla*	DQ536428.1
	*Scomberomorus niphonius*	NC_016420
	*Scomberomorus concolor*	KX925518.1
	*Scomberomorus guttatus*	this study
	*Scomberomorus commerson*	this study
	*Scomberomorus maculatus*	OP056995.2
	*Scomberomorus munroi*	NC_021390.1
	*Scomberomorus semifasciatus*	NC_021391.1
	*Scomberomorus sierra*	KX925517.1
*Thunnus*	*Thunnus alalunga*	NC_005317
	*Thunnus albacares*	NC_014061
	*Thunnus atlanticus*	NC_025519.1
	*Thunnus maccoyii*	NC_014101
	*Thunnus obesus*	NC_014059
	*Thunnus orientalis*	NC_008455
	*Thunnus thynnus*	NC_014052
	*Thunnus tonggol*	HQ425780
*Acanthocybium*	*Acanthocybium solandri*	NC_067731.1
*Sarda*	*Sarda orientalis*	AP012949.1
*Gymnosarda*	*Gymnosarda unicolor*	AP012510.1

**Table 2:** Composition of the mitochondrial genome sequences of *S. guttatus* (*S.g*) and *S. commerson* (*S.c*).

Sequence	Position		Length		Start Codon		Stop Codon		Spacing Nucleotide Count		Coding Strand	
	*S.g*	*S.c*	*S.g*	*S.c*	*S.g*	*S.c*	*S.g*	*S.c*	*S.g*	*S.c*	*S.g*	*S.c*
*Cytb*	1–1141	-	1141	-	ATG		T--	-	4	-	H	
*tRNA-Thr*	1142–1215	1142–1213	74	72					0	-	H	
*tRNA-Pro*	1215–1284	1213–1282	70	-					−1	-	L	
*D-loop*	1285–2141	1283–2137	857	855					0	-	H	
*tRNA-Phe*	2142–2209	2138–2205	68	-					0	-	H	
*s-rRNA*	2210–3176	2206–3162	967	957					0	-	H	
*tRNA-Val*	3177–3248	3163–3234	72	-					0	-	H	
*l-rRNA*	3249–4945	3235–4927	1697	1693					0	-	H	
*tRNA-Leu*	4946–5019	4928–5001	74	-					0	-	H	
*ND1*	5020–5994	5002–5976	975	-	ATG		TAA	-	0	-	H	
*tRNA-Ile*	5999–6069	5981–6051	71	-					4	-	H	
*tRNA-Gln*	6069–6139	6051–6121	71	-					−1	-	L	
*tRNA-Met*	6139–6207	6121–6189	69	-					−1	-	H	
*ND2*	6208–7253	6190–7235	1046	-	ATG		TA-	-	0	-	H	
*tRNA-Trp*	7254–7326	7236–7308	73	-					0	-	H	
*tRNA-Ala*	7328–7396	7310–7378	69	-					1	-	L	
*tRNA-Asn*	7398–7470	7380–7452	73	-					1	-	L	
*OL(rep_origin)*	7471–7503	7453–7486	33	34					0	-	H	
*tRNA-Cys*	7504–7569	7487–7552	66	-					0	-	L	
*tRNA-Tyr*	7570–7636	7553–7620	67	68					0	-	L	
*COI*	7638–9185	7622–9181	1548	1560	GTG		AGA	AGG	1	-	H	
*tRNA-Ser*	9188–9259	9173–9244	72	-					2	−9	L	
*tRNA-Asp*	9263–9335	9248–9320	73	-					3	-	H	
*COII*	9344–10,034	9329–10,019	691	-	ATG		T--	-	8	-	H	
*tRNA-Lys*	10,035–10,108	10,020–10,093	74	-					0	-	H	
*ATP8*	10,110–10,277	10,095–10,262	168	-	ATG		TAA	-	1	-	H	
*ATP6*	10,268–10,951	10,253–10,936	684	-	ATG		TAA	-	−10	-	H	
*COIII*	10,971–11,755	10,955–11,739	785	-	ATG		TA-	-	19	18	H	
*tRNA-Gly*	11,756–11,827	11,740–11,811	72	-					0	-	H	
*ND3*	11,828–12,176	11,812–12,160	349	-	ATG		T--	-	0	-	H	
*tRNA-Arg*	12,177–12,245	12,161–12,229	69	-					0	-	H	
*ND4L*	12,246–12,542	12,230–12,526	297	-	ATG		TAA	-	0	-	H	
*ND4*	12,536–13,916	12,520–13,900	1381	-	ATG		T--	-	−7	-	H	
*tRNA-His*	13,917–13,987	13,901–13,970	71	70					0	-	H	
*tRNA-Ser*	13,988–14,055	13,971–14,038	68	-					0	-	H	
*tRNA-Leu*	14,060–14,132	14,043–14,115	73	-					4	-	H	
*ND5*	14,133–15,971	14,116–15,954	1839	-	ATG		TAA	-	0	-	H	
*ND6*	15,968–16,489	15,951–16,472	522	-	ATG		TAA	TAG	−4	-	L	
*tRNA-Glu*	16,490–16,558	16,473–16,541	69	-					0	-	L	

**Table 3:** Base composition of gene segments in *S. guttatus* and *S. commerson*.

Sequence	A%		T%		G%		C%		A + T%		G + C%		Total	
	*S.g*	*S.c*	*S.g*	*S.c*	*S.g*	*S.c*	*S.g*	*S.c*	*S.g*	*S.c*	*S.g*	*S.c*	*S.g*	*S.c*
mtDNA complete sequence	28.5	28.1	26.6	25.3	16	16.2	28.9	30.3	55.1	53.5	44.9	46.5	16,562	16,545
Protein-coding genes	26.2	25.7	28.6	27.1	15.3	15.6	29.9	31.6	54.8	52.8	45.2	47.3	11,426	11,438
tRNA genes	27.5	27	27.2	26.9	23.7	24.4	21.6	21.7	54.7	53.9	45.3	46	1558	1556
rRNA gene	31.3	32.2	22.5	21.5	21.1	21	25.1	25.3	53.8	53.7	46.2	46.3	2664	2650
Control region	32.2	31.1	30.9	32.1	16	14.9	20.9	22	63.1	63.1	36.9	36.8	857	855

PS: *S.g*: *S. guttatus*. *S.c*: *S. commerson*.

## Data Availability

The complete mitogenomes of *S. guttatus* and *S. commerson* has been published inthe Genbank public database with accession number pp437201 and pp437202.
